# Type I Interferon-Related Gene Expression and Laboratory Abnormalities in Acute Infection Are Associated with Long COVID Symptom Burden

**DOI:** 10.3390/jcm14217875

**Published:** 2025-11-06

**Authors:** Mary Emmanouil, Vasiliki E. Georgakopoulou, Konstantinos Drougkas, Panagiotis Lembessis, Charalampos Skarlis, Aikaterini Gkoufa, Nikolaos V. Sipsas, Clio P. Mavragani

**Affiliations:** 1 National Influenza Reference Laboratory of Southern Greece, Hellenic Pasteur Institute, 11521 Athens, Greece; emmanouilm@pasteur.gr; 2 Department of Pathophysiology, Laiko General Hospital, National and Kapodistrian University of Athens, 11527 Athens, Greece; vaso_georgakopoulou@hotmail.com (V.E.G.);; 3 Infectious Diseases-COVID-19 Unit, Laiko General Hospital, 11527 Athens, Greece; katergouf@yahoo.gr; 4 Department of Physiology, School of Medicine, National and Kapodistrian University of Athens, 11527 Athens, Greece

**Keywords:** long COVID, acute disease, hematological findings, type I IFN

## Abstract

**Background:** Long COVID—defined as the persistence of symptoms or the development of new symptoms beyond four weeks after acute SARS-CoV-2 infection—affects an estimated 10–30% of individuals recovering from COVID-19, posing a significant public health burden. Emerging evidence suggests that type I interferons (IFNs) (a critical group of cytokines in the antiviral defense) and hematologic alterations, such as lymphopenia and elevated inflammatory markers, are linked to both the severity of acute COVID-19 and the likelihood of developing long-term symptoms. The aim of this study is to explore the association between type I IFN signatures and long COVID. A second aim is to examine the relationship between laboratory findings during acute infection and long COVID. **Methods**: The study included 61 patients investigated for the presence of long COVID symptoms 16.5 ±1.5 months after acute infection. Patients were divided into two groups of higher symptom burden of long COVID and those with milder symptoms based on demographic, laboratory, and clinical data as well as type I IFN-inducible gene expression (MX-1, IFIT-1, and IFI-44) measured in peripheral blood by real-time PCR. Data collected during acute infection were recorded. Peripheral blood samples were collected during the acute phase of infection, within the first 48 h of hospital admission. IFN-inducible gene expression was measured prospectively at that time, and RNA was extracted immediately for subsequent analysis. **Results**: History of intubation emerged as a significant associated factor of severe long COVID, with 75% of intubated patients reporting >8 persistent symptoms approximately 16 months post-infection. Higher white blood cell (WBC) and neutrophil counts but lower eosinophil and monocyte counts in acute infection were found to be associated with a high burden of long COVID symptoms. Interestingly, absolute monocyte count was found to independently correlate with higher long COVID symptom burden. Lactate dehydrogenase (LDH) and serum glutamic-oxaloacetic transaminase (SGOT) also differed significantly between groups, with higher levels correlating with a high burden of long COVID symptoms. Notably, MX-1 transcript levels in peripheral blood at the time of acute infection were reduced in patients with a high burden of long COVID symptoms, suggesting that dysregulated immune responses during the acute phase may contribute to persistent symptoms. **Conclusions**: These findings suggest the potential association of hematological and immune markers with long COVID severity, as well as the importance of monitoring these parameters to identify at-risk patients for early interventions.

## 1. Introduction

The severe acute respiratory syndrome coronavirus 2 (SARS-CoV-2) that caused the coronavirus disease 2019 (COVID-19) pandemic has not only resulted in widespread acute illness, but also prolonged (more than four weeks) health complications known as long COVID or post-acute sequelae of SARS-CoV-2 infection (PASC) [1]. Characterized by persistent symptoms and new health issues that develop after the acute phase of the infection, long-term COVID affects an estimated 10–30% of individuals recovering from COVID-19, presenting a significant public health burden. Beyond its clinical manifestations, long COVID poses a substantial socioeconomic and public health burden. It contributes to reduced quality of life, prolonged work absences, increased healthcare utilization, and significant economic costs, particularly in working-age populations [2].

Emerging evidence suggests that the immune response during the acute phase of COVID-19 plays a crucial role in determining the trajectory towards long-term COVID [3]. It has been previously shown that type I interferons (IFNs), a critical group of cytokines in the antiviral defense, significantly influence disease outcomes, with weak type I IFN responses being linked to worse COVID-19 acute and chronic symptoms [4,5].

Additionally, studies have linked hematologic alterations during the acute phase, such as changes in white blood cell counts, lymphopenia, and elevated inflammatory markers, to the severity of COVID-19 and may predict the likelihood of developing long-term COVID. Importantly, the immune response during acute infection is characterized by rapid antiviral activity, whereas the post-acute phase often involves persistent immune dysregulation, which may drive chronic inflammation and symptom persistence [6].

The goal of the present study is to investigate whether clinical and laboratory features, as well as type I IFN-inducible gene expression levels measured in peripheral blood during COVID-19 acute infection, can be associated with the occurrence of long-term COVID symptoms. By identifying these correlations, this study aims to contribute to the development of associated factors for long COVID, enhancing early identification and intervention strategies.

To our knowledge, this is one of the few studies to investigate IFN-inducible gene expression during the acute phase as a potential biomarker for long COVID, offering novel insight into early immune signatures associated with persistent symptoms.

## 2. Materials and Methods

### 2.1. Study Population

Sixty-one patients with a history of acute COVID-19 infection requiring hospitalization at the COVID-19 unit (Laiko General Hospital) from October 2020 to February 2021 (predominance of the alpha variant) were evaluated for the presence of long COVID symptoms 16.5 months (range: 15–18 months) after discharge. Patients were consecutively enrolled during the study period to minimize selection bias and ensure a representative sampling of hospitalized COVID-19 cases.

Clinical and laboratory data, as well as type I IFN-inducible gene expression in peripheral blood [interferon-induced GTP-binding protein Mx1 (MX-1), interferon-induced protein with tetratricopeptide repeats 1 (IFIT-1), and interferon-induced protein 44-like (IFI-44), were available in the setting of a previously published study from our group [6]. A control group of patients (*n* = 30) who were not hospitalized after COVID-19 infection was also evaluated for long COVID symptoms. A non-hospitalized control group (*n* = 30) was included to provide a reference distribution of symptom burden in individuals with mild COVID-19. This control group was used solely to derive the symptom threshold for defining high long COVID symptom burden. Control participants were matched by age and sex distribution to the study group to minimize demographic confounding.

### 2.2. Data Collection

Patients enrolled in the study were asked to report long COVID symptoms during in-person or telephone interviews conducted from 1 June 2022 to 6 July 2022, based on the Newcastle post-COVID syndrome Follow-Up Screening Questionnaire [7] (Supplementary File S1). The questionnaire was first translated into Greek by a professional translation company. The translated score was then back-translated into English by clinicians who were proficient in the language. The clinicians determined the final version of the translated score. The translated questionnaire underwent validation to ensure linguistic and conceptual equivalence. Face and content validity were confirmed through expert review and pilot testing in a representative sample of participants.

Long COVID symptoms were documented for hospitalized and control group patients and further classified into general, respiratory, cardiovascular, musculoskeletal, ear, nose, and throat (ENT), and neuropsychiatric symptoms [8]. According to the number of symptoms reported, our study population was divided into two subsets: Group A: long COVID with >8 symptoms (*n* = 12) (high symptom burden); Group B: long COVID with ≤8 symptoms (*n* = 49) (low symptom burden). The range of symptoms reported by the non-hospitalized patient control group was between 0 and 7 symptoms (1.07 ± 1.8). In line with established guidelines [9], high-burden long COVID symptom positivity was defined as a score exceeding the mean of the controls by four standard deviations (mean ± 4SD), ensuring a highly specific criterion for differentiating high from low long COVID symptom burden groups of individuals.

All participants were infected during the early wave of the pandemic (October 2020–February 2021), when the alpha variant was predominant. This period preceded the initiation of the national COVID-19 vaccination program; therefore, none of the participants had been vaccinated prior to infection. Reinfections were not documented during the follow-up period. Comorbidities were recorded at baseline, and their distribution did not differ significantly between groups with high and low long COVID symptom burden (Table 1).

### 2.3. Statistical Analysis

Descriptive statistics were used to summarize the data. The normality of continuous variables was tested using the Shapiro–Wilk test before applying parametric tests. Non-parametric alternatives were considered when data did not meet normality assumptions. Continuous variables were expressed as mean ± standard deviation (SD) and categorical variables as frequencies and percentages. Comparisons between groups were made using the independent *t*-test for continuous variables and the chi-square test for categorical variables. A *p*-value of less than 0.05 was considered statistically significant. Statistical analyses were conducted using SPSS version 26 (IBM Corp., Armonk, NY, USA).

### 2.4. Ethical Considerations

The study protocol was reviewed and approved by the Institutional Review Board of Laiko General Hospital (protocol number: 18954-14/12/2020). Informed consent was obtained from all participants.

## 3. Results

### 3.1. Demographics and Distribution of Long COVID Symptoms According to Organ Involvement in Study Participants and Controls

**Figure 1:**
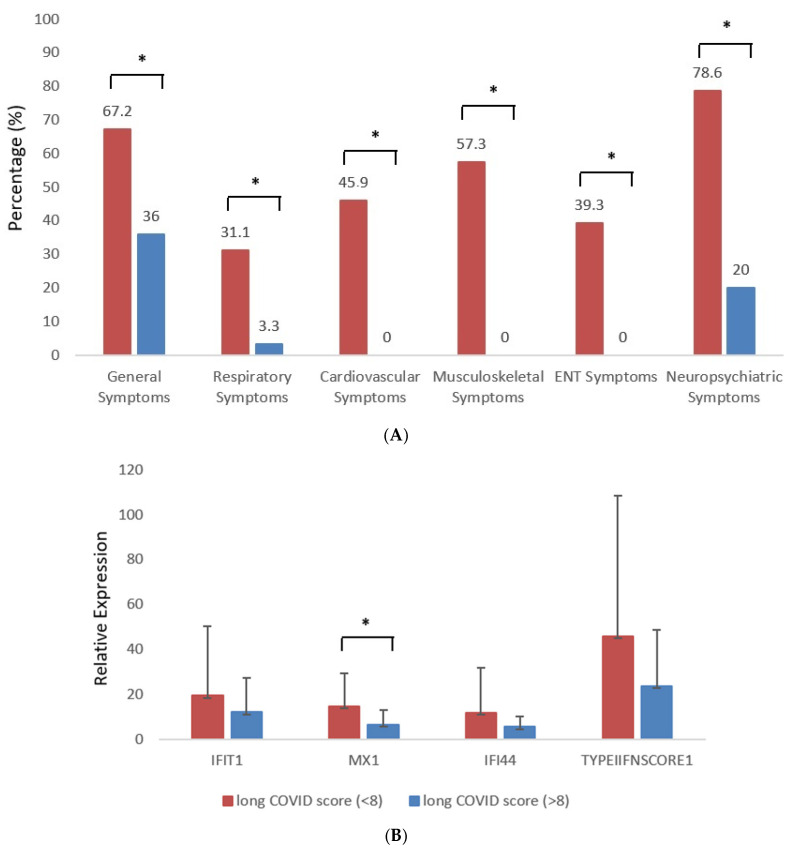
(**A**) Assessment of long COVID symptom burden documented for hospitalized and control group patients, classified into general, respiratory, cardiovascular, musculoskeletal, ear, nose, and throat (ENT), and neuropsychiatric symptoms (* indicate *p* value < 0.05). (**B**) Comparison of type I IFN-inducible gene expression (MX-1) during acute infection between patients who subsequently developed high vs. low long COVID symptom burden (* indicate *p* value < 0.05). Red = high-symptom-burden group; blue = low-symptom-burden group.

A total of 61 patients (57.3% males) with a mean age of 58.4 ± 13.4 years were included in the study, compared to a control group (*n* = 30) of non-hospitalized patients after COVID-19 infection, which included 36,6% males with a mean age of 48.6 ± 15.5 years. As displayed in Figure 1A, neuropsychiatric and general symptoms, along with musculoskeletal symptoms, were the most prevalent complaints reported by 78.6%, 67.2%, and 57.3% of study participants, respectively. Neuropsychiatric symptoms included difficulty with sleeping, nightmares, memory, and concentration problems, headaches, nausea, dizziness, bad mood, anxiety, and depression, while general symptoms involved fatigue, malaise, problems in physical status recovery, weakness, recurrent fever, and weight loss or gain. Cardiovascular and ENT symptoms were present in 45.9% and 39.3% of patients, respectively. Of note, respiratory symptoms were less prominent, affecting 31.1% of these patients. As shown in Figure 1B, MX-1 transcript levels during acute infection were significantly lower in patients with high long COVID symptom burden compared with those with low symptom burden (*p* = 0.04).

### 3.2. Association of Long COVID Symptom Burden with Clinical, Hematological, and Serological Variables at Baseline

Among hospitalized patients, 12/61 (19.7%) were classified as having a high symptom burden (>8 symptoms) and 49/61 (80.3%) as having a low symptom burden (≤8 symptoms).

As shown in Table 1, a history of intubation was present in 9/12 (75%) patients with high symptom burden compared to 12/49 (25%) in the low-symptom-burden group (*p* = 0.004). There was no significant difference in mean age between patients with high (58.6 ± 12.1 years, *n* = 12) and low symptom burden (57.9 ± 13.8 years, *n* = 49). A trend toward a higher symptom burden in males was observed, with 8/12 (69.2%) males in the high-symptom-burden group vs. 19/49 (38.8%) males in the low-symptom-burden group (*p* = 0.06). In addition, no statistically significant differences were observed between the two groups regarding the presence of comorbidities, such as diabetes, cardiovascular disease, autoimmune disease/hypothyroidism, and chronic respiratory disease (all *p* > 0.05).

Relating to hematological variables, a higher WBC count (7.4 vs. 5.9 K/μL, *p* = 0.04) and absolute neutrophil count (7722 vs. 6904/μL, *p* = 0.02), alongside a lower absolute monocyte count (511 vs. 948/μL, *p* = 0.009) and absolute eosinophil count (158 vs. 747/μL, *p* = 0.03) at baseline, were linked to higher long COVID symptom burden. Though non-significant, a trend of raised inflammatory markers [erythrocyte sedimentation rate (ESR), C-reactive protein (CRP), and ferritin] was also detected in the high-symptom-burden group. Of interest, markers of tissue damage, including serum glutamic-oxaloacetic transaminase (SGOT) and lactate dehydrogenase (LDH) levels, were significantly higher in patients with high long COVID burden compared to their counterparts with lower numbers of symptoms (*p*-values of 0.005 and 0.002, respectively).

Given that type I IFN pathways have been consistently detected as determinants of COVID-19 severity [10,11], we sought to explore whether type I IFN-inducible gene expression in peripheral blood at the time of active infection could be related to long COVID symptom burden. As shown in Figure 1, among the interferon-stimulated genes examined (MX1, IFIT1, and IFI44), only MX1 transcript levels were significantly lower in patients with a high long COVID symptom burden (6.4 ± 6.4 vs. 14.7 ± 14.8; *p*-value: 0.04). IFIT1 and IFI44 showed inconsistent and non-significant trends. No composite interferon score was calculated.

Furthermore, we performed a multivariate logistic regression analysis including all variables that were significant in the univariate analysis, specifically intubation, SGOT, LDH, MX-1, absolute monocyte count, absolute eosinophil count, absolute neutrophil count, and WBC, as shown in Table 1. Among those variables, only absolute monocyte counts turned out to be independently associated with a severity score >8 (*p* = 0.008 OR 95% CI (0.407–0.874)). These findings suggest that absolute monocyte count may serve as an independent indicator of increased long COVID severity.

## 4. Discussion

In the present study, we report that distinct clinical and laboratory profiles, as well as MX-1 expression levels in peripheral blood in the acute phase of COVID-19 infection requiring hospitalization, could serve as potential associated factors for the number of long COVID symptoms developed approximately 16 months following acute infection. Thus, the history of intubation, elevated WBC and neutrophil counts, and higher inflammatory markers and SGOT/LDH serum levels were shown to be related to an increased number of long COVID symptoms. Moreover, lower eosinophil and monocyte counts, together with downregulated MX-1 mRNA expression in peripheral blood at the time of acute infection, were also linked to higher long COVID symptom burden. Our analysis further underscores the potential role of absolute monocyte count as an independent indicator of long COVID symptom severity, warranting further investigation in larger cohorts.

Long COVID refers to a range of symptoms that persist for more than 4 weeks following the acute phase of COVID-19. These symptoms can occur in individuals regardless of the severity of their initial infection and may include fatigue, shortness of breath, cognitive dysfunction (“brain fog”), chest pain, joint or muscle aches, and palpitations. In many cases, symptoms fluctuate or relapse over time, significantly affecting daily functioning and quality of life [8].

Long COVID is characterized by persistent inflammation and immune dysregulation. Common findings include anemia, leukocytosis, neutrophilia, lymphopenia, and elevated inflammatory markers such as ferritin, CRP, and D-dimers, indicating ongoing inflammation and coagulation disturbances [12,13,14].

Lower eosinophil and monocyte counts during the acute phase may reflect a state of innate immune suppression and cellular redistribution. Eosinopenia is common in acute viral illness and can be driven by stress-induced glucocorticoids and catecholamines, type I IFN-mediated trafficking, and accelerated homing of eosinophils to inflamed tissues, leading to transient depletion in peripheral blood [15]. Monocytopenia may similarly result from emergency myelopoiesis with a neutrophil-biased output, preferential recruitment of circulating monocytes to the lung, and functional reprogramming toward HLA-DR^low/immature phenotypes that blunt antigen presentation [16,17,18]. Collectively, these changes could impair early antiviral clearance and resolution pathways, favoring persistent tissue injury and the downstream symptomatology of long COVID.

Elevated LDH and SGOT levels during acute infection were also associated with more severe long COVID symptoms. A meta-analysis by Fialek et al. linked high LDH levels to poor COVID-19 outcomes [19], while Udeh et al. associated elevated LDH with persistent respiratory symptoms [20]. SGOT elevation, an indicator of multi-organ inflammation, has been linked to disease severity and prolonged symptoms, particularly in hospitalized patients [21,22,23,24,25].

While previous studies have used composite type I interferon scores derived from multiple interferon-stimulated genes, our analysis was restricted to three genes. Of these, only MX1 showed a statistically significant association with symptom burden, whereas IFIT1 and IFI44 exhibited non-significant and inconsistent patterns. Therefore, our results should not be interpreted as evidence of a global interferon signature. Rather, they point to potential involvement of MX1 expression specifically, warranting further investigation in studies with more comprehensive interferon gene panels.

Type I and III IFNs play a crucial antiviral role but are targeted by viral proteins (Nsp1, Nsp3, and ORF6), enabling unchecked viral replication [26]. Genetic predisposition, including IFN-related gene errors and autoantibodies against type I IFNs, worsens disease severity, particularly in older adults, males, and individuals with comorbidities [6,10,27,28,29].

Emerging evidence links IFN dysregulation to long COVID. Ghorra et al. [30] reported chronic immune activation with reduced type I IFN production, likely due to immune exhaustion or epigenetic changes, potentially increasing susceptibility to secondary infections. Wang et al. [31] found an absence of significant IFN-γ responses in long COVID patients, suggesting that alternative cytokines drive symptom persistence, highlighting IFN pathway modulation as a potential treatment.

Gómez-Carballa et al. [32] observed that while type I and III IFN signatures were elevated in acute severe COVID-19, they declined in long COVID, suggesting cytokine exhaustion or persistent immune suppression. Our study also found downregulated IFN signatures during acute infection, correlating with severe long COVID.

Neurocognitive symptoms such as brain fog may be linked to IFN-I dysregulation. Vavougios et al. [33] suggested that persistent IFN-I activation disrupts CNS homeostasis, resembling neurodegenerative processes like Alzheimer’s. Age-related differences in IFN-I signaling were also reported, with adolescents exhibiting elevated IFN-β, IFN-ε, and IFN-ω, particularly in those with neurological symptoms, while younger children showed decreased levels [34,35].

Severe COVID-19 and long COVID are also linked to increased expression of cGAS and STING genes, along with elevated plasma IFN-α. Activation of this pathway drives persistent autoinflammatory responses, correlating with high IFN-α levels in acute and severe long COVID patients [36].

While our study provides important insights, several limitations must be acknowledged. The relatively small sample size and the specific cohort (patients from a single hospital in Greece) may limit the generalizability of our findings. Larger, more diverse cohorts are necessary to validate our results and ensure broader applicability. The follow-up period varied among patients, potentially influencing the consistency of the findings. Longitudinal studies with standardized follow-up intervals are needed to better understand the progression and persistence of long COVID symptoms. The presence of preexisting conditions and varying treatment regimens during acute COVID-19 were not fully controlled for, which could confound the observed associations between hematologic parameters, IFN scores, and long COVID symptoms. While we identified associations between specific biomarkers and long COVID, the specificity and sensitivity of these markers for predicting long COVID require further investigation. Additionally, the reliance on patient self-reporting for long COVID symptoms may introduce recall bias, which could affect the accuracy of symptom characterization and group classification. Future studies should aim to develop a comprehensive biomarker profile for early identification and management of long COVID. In addition, future studies should aim to validate these findings in larger, multicenter cohorts and through longitudinal designs, which would allow for more robust evaluation of temporal associations and external generalizability.

## 5. Conclusions

This study underscores the importance of hematologic and immune parameters in the pathogenesis of long COVID. Elevated type I interferon-related gene expression and hematologic alterations during acute COVID-19 may be associated with more severe long COVID symptoms, highlighting potential biomarkers for identifying at-risk patients. However, further research with larger, diverse cohorts and standardized methodologies is essential to confirm these findings and develop effective therapeutic strategies for long COVID management.

## Figures and Tables

**Table 1:** Comparisons of clinical outcomes and laboratory findings between patients with high long COVID symptom burden (>8 symptoms) and low long COVID burden (≤8 symptoms).

Parameter	Long COVID (Score ≤ 8)	Long COVID (Score > 8)	*p*-Value
Age	58.5 ± 12.6	56.8 ± 15.3	0.96
Female Gender (%)	69.2	30.8	0.06
Comorbidities—Diabetes	2 (4.2%)	2 (15.4%)	0.196
Comorbidities—Cardiovascular Disease	24 (50.0%)	3 (23.1%)	0.118
Comorbidities—Autoimmune Disease/Hypothyroidism	7 (14.6%)	5 (38.5%)	0.108
Comorbidities—Chronic Respiratory Disease	8 (16.7%)	5 (38.5%)	0.126
Intubation (%)	25	75	0.004 *
High Disease Severity (%)	22.7	77.3	0.33
Hemoglobin (g/L)	13.5 ± 1.7	13.1 ± 1.4	0.35
Platelet Count (×10^3^/μL)	221 ± 97	377 ± 531	0.97
WBC (×10^3^/μL) (mean ± SD)	5.9 ± 2.4	7.4 ± 2.7	0.04 *
Absolute Neutrophil Count (×10^3^/μL)	6.90 ± 1.13	7.72 ± 1.13	0.02 *
Absolute Lymphocyte Count (×10^3^/μL)	2.24 ± 0.95	1.72 ± 0.98	0.08
Absolute Monocyte Count (×10^3^/μL)	0.94 ± 0.15	0.51 ± 0.15	0.009 *
Absolute Eosinophil Count (×10^3^/μL)	0.74 ± 0.13	0.15 ± 0.31	0.03 *
Troponin (pg/mL)	13.8 ± 25.8	9.5 ± 5.7	0.74
Creatinine (mg/dL)	1.2 ± 2.2	0.7 ± 0.1	0.14
Urea (mg/dL)	37.3 ± 28.7	30.5 ± 9.6	0.52
SGOT (U/L)	31.3 ± 17.2	47.2 ± 19.7	**0.005** *
SGPT (U/L)	32.4 ± 32.7	44.3 ± 22.9	0.35
GGT (U/L)	39.2 ± 33.1	47 ± 34.4	0.26
Alkaline Phosphatase (U/L)	68.2 ± 28.2	55.3 ± 17	0.07
CK (U/L)	143.8 ± 175	218.1 ± 231.8	0.18
LDH (U/L)	264.7 ± 94	397.2 ± 143.6	0.002 *
D-dimers (µg/mL)	0.97 ± 0.78	0.74 ± 0.4	0.44
ESR (mm/1 h)	46 ± 33	80 ± 26	0.08
CRP (mg/L)	43.8 ± 50.7	91.3 ± 96.8	0.08
Ferritin (ng/mL)	517 ± 583	699 ± 499	0.10
Fibrinogen (mg/dL)	527 ± 158	573 ± 176	0.31

* indicate *p* value <0.05.

## Data Availability

The original contributions presented in the study are included in the article/Supplementary Materials, and further inquiries can be directed to the corresponding author.

## Supplementary Materials

The following supporting information can be downloaded at https://www.mdpi.com/article/10.3390/jcm14217875/s1, File S1: Long COVID pre-assessment questionnaire.

## References

[1] Nalbandian A., Sehgal K., Gupta A., Madhavan M.V., McGroder C., Stevens J.S., Cook J.R., Nordvig A.S., Shalev D., Sehrawat T.S. (2021). Post-acute COVID-19 syndrome. Nat. Med..

[2] Davis H.E., Assaf G.S., McCorkell L., Wei H., Low R.J., Re’em Y., Redfield S., Austin J.P., Akrami A. (2021). Characterizing long COVID in an international cohort: 7 months of symptoms and their impact. EClinicalMedicine.

[3] Peluso M.J., Deeks S.G. (2024). Mechanisms of long COVID and the path toward therapeutics. Cell.

[4] Su H.C., Jing H., Zhang Y., Casanova J.L., Members of the COVID Human Genetic Effort (2023). Interfering with Interferons: A Critical Mechanism for Critical COVID-19 Pneumonia. Annu. Rev. Immunol..

[5] Hadjadj J., Yatim N., Barnabei L., Corneau A., Boussier J., Smith N., Péré H., Charbit B., Bondet V., Chenevier-Gobeaux C. (2020). Impaired type I interferon activity and inflammatory responses in severe COVID-19 patients. Science.

[6] Georgakopoulou V.E., Lembessis P., Skarlis C., Gkoufa A., Sipsas N.V., Mavragani C.P. (2022). Hematological Abnormalities in COVID-19 Disease: Association with Type I Interferon Pathway Activation and Disease Outcomes. Front. Med..

[7] Penny’s Hill Practice (2021). Post-COVID Newcastle Screening Tool.

[8] Tsilingiris D., Vallianou N.G., Karampela I., Christodoulatos G.S., Papavasileiou G., Petropoulou D., Magkos F., Dalamaga M. (2023). Laboratory Findings and Biomarkers in Long COVID: What Do We Know So Far? Insights into Epidemiology, Pathogenesis, Therapeutic Perspectives and Challenges. Int. J. Mol. Sci..

[9] Streiner D., Norman G., Cairney J. (2015). Health Measurement Scales: A Practical Guide to Their Development and Use.

[10] Bencze D., Fekete T., Pázmándi K. (2022). Correlation between Type I Interferon Associated Factors and COVID-19 Severity. Int. J. Mol. Sci..

[11] Montenegro A.F.L., Clementino M.A.F., Yaochite J.N.U. (2024). Type I interferon pathway genetic variants in severe COVID-19. Virus Res..

[12] Lechuga G.C., Morel C.M., De-Simone S.G. (2023). Hematological alterations associated with long COVID-19. Front. Physiol..

[13] Mohiuddin Chowdhury A.T.M., Karim M.R., Ali M.A., Islam J., Li Y., He S. (2021). Clinical Characteristics and the Long-Term Post-recovery Manifestations of the COVID-19 Patients—A Prospective Multicenter Cross-Sectional Study. Front. Med..

[14] Radkhah H., Omidali M., Hejrati A., Bahri R.A., Arefi S., Behzadi A., Eslami M., Khadembashiri M., Khadembashiri M., Najafirashed M. (2023). Correlations of Long COVID Symptoms and Inflammatory Markers of Complete Blood Count (CBC): A Cross-sectional Study. J. Community Hosp. Intern. Med. Perspect..

[15] Hong S.G., Sato N., Legrand F., Gadkari M., Makiya M., Stokes K., Howe K.N., Yu S.J., Linde N.S., Clevenger R.R. (2020). Glucocorticoid-Induced Eosinopenia Results from CXCR4-Dependent Bone Marrow Migration. Blood.

[16] Liao M., Liu Y., Yuan J., Wen Y., Xu G., Zhao J., Cheng L., Li J., Wang X., Wang F. (2020). Single-Cell Landscape of Bronchoalveolar Immune Cells in Patients with COVID-19. Nat. Med..

[17] Silvin A., Chapuis N., Dunsmore G., Goubet A.G., Dubuisson A., Derosa L., Almire C., Hénon C., Kosmider O., Droin N. (2020). Elevated Calprotectin and Abnormal Myeloid Cell Subsets Discriminate Severe from Mild COVID-19. Cell.

[18] Schulte-Schrepping J., Reusch N., Paclik D., Baßler K., Schlickeiser S., Zhang B., Krämer B., Krammer T., Brumhard S., Bonaguro L. (2020). Severe COVID-19 Is Marked by a Dysregulated Myeloid Cell Compartment. Cell.

[19] Fialek B., Pruc M., Smereka J., Jas R., Rahnama-Hezavah M., Denegri A., Szarpak A., Jaguszewski M.J., Peacock F.W., Szarpak L. (2022). Diagnostic value of lactate dehydrogenase in COVID-19: A systematic review and meta-analysis. Cardiol. J..

[20] Udeh R., Utrero-Rico A., Dolja-Gore X., Rahmati M., McEVoy M., Kenna T. (2023). Lactate dehydrogenase contribution to symptom persistence in long COVID: A pooled analysis. Rev. Med. Virol..

[21] Georgakopoulou V.E., Bali T., Adamantou M., Asimakopoulou S., Makrodimitri S., Samara S., Triantafyllou M., Voutsinas P.M., Eliadi I., Karamanakos G. (2022). Acute hepatitis and liver injury in hospitalized patients with COVID-19 infection. Exp. Ther. Med..

[22] Cholongitas E., Bali T., Georgakopoulou V.E., Kamiliou A., Vergos I., Makrodimitri S., Samara S., Triantafylou M., Basoulis D., Eliadi I. (2022). Comparison of liver function test- and inflammation-based prognostic scores for coronavirus disease 2019: A single center study. Eur. J. Gastroenterol. Hepatol..

[23] Cholongitas E., Bali T., Georgakopoulou V.E., Giannakodimos A., Gyftopoulos A., Georgilaki V., Gerogiannis D., Basoulis D., Eliadi I., Karamanakos G. (2022). Prevalence of abnormal liver biochemistry and its impact on COVID-19 patients’ outcomes: A single-center Greek study. Ann. Gastroenterol..

[24] Bali T., Georgakopoulou V.E., Kamiliou A., Vergos I., Adamantou M., Vlachos S., Ermidis G., Sipsas N.V., Samarkos M., Cholongitas E. (2023). Abnormal liver function tests and coronavirus disease 2019: A close relationship. J. Viral. Hepat..

[25] de Lima I.C., de Menezes D.C., Uesugi J.H.E., Bichara C.N.C., da Costa Vasconcelos P.F., Quaresma J.A.S., Falcão L.F.M. (2023). Liver Function in Patients with Long-Term Coronavirus Disease 2019 of up to 20 Months: A Cross-Sectional Study. Int. J. Environ. Res. Public Health.

[26] Kim Y.M., Shin E.C. (2021). Type I and III interferon responses in SARS-CoV-2 infection. Exp. Mol. Med..

[27] Mavragani C.P., Skarlis C., Kostopoulos I.V., Maratou E., Moutsatsou P., Terpos E., Tsitsilonis O.E., Dimopoulos M.A., Sfikakis P.P. (2022). Distinct type I interferon responses between younger women and older men contribute to the variability of COVID-19 outcomes: Hypothesis generating insights from COVID-19 convalescent individuals. Cytokine.

[28] Zhang Q., Bastard P., Liu Z., Le Pen J., Moncada-Velez M., Chen J., Ogishi M., Sabli I.K.D., Hodeib S., Korol C. (2020). Inborn errors of type I IFN immunity in patients with life-threatening COVID-19. Science.

[29] van der Made C.I., Simons A., Schuurs-Hoeijmakers J., van den Heuvel G., Mantere T., Kersten S., van Deuren R.C., Steehouwer M., van Reijmersdal S.V., Jaeger M. (2020). Presence of Genetic Variants Among Young Men with Severe COVID-19. JAMA.

[30] Ghorra N., Popotas A., Besse-Hammer T., Rogiers A., Corazza F., Nagant C. (2024). Cytokine Profile in Patients with Postacute Sequelae of COVID-19. Viral Immunol..

[31] Wang Y., Guo L., Cui D., Zhang H., Zhang Q., Ren L., Wang G., Zhang X., Huang T., Chen L. (2024). Immune Responses in Discharged COVID-19 Patients with and Without Long COVID Symptoms. Open Forum. Infect. Dis..

[32] Gómez-Carballa A., Pischedda S., Pardo-Seco J., Gómez-Rial J., Martinón-Torres F., Salas A. (2024). Interferon gene expression declines over time post-COVID infection and in long COVID patients. Infect. Dis..

[33] Vavougios G.D., Tseriotis V.S., Liampas A., Mavridis T., de Erausquin G.A., Hadjigeorgiou G. (2024). Type I interferon signaling, cognition and neurodegeneration following COVID-19: Update on a mechanistic pathogenetic model with implications for Alzheimer’s disease. Front. Hum. Neurosci..

[34] Fracella M., Mancino E., Nenna R., Virgillito C., Frasca F., D’Auria A., Sorrentino L., Petrarca L., La Regina D., Matera L. (2024). Age-related transcript changes in type I interferon signaling in children and adolescents with long COVID. Eur. J. Immunol..

[35] Gross R.S., Thaweethai T., Kleinman L.C., Snowden J.N., Rosenzweig E.B., Milner J.D., Tantisira K.G., Rhee K.E., Jernigan T.L., Kinser P.A. (2024). Characterizing Long COVID in Children and Adolescents. JAMA.

[36] Queiroz M.A.F., Brito W.R.D.S., Pereira K.A.S., Pereira L.M.S., Amoras E.D.S.G., Lima S.S., Santos E.F.D., Costa F.P.D., Sarges K.M.L., Cantanhede M.H.D. (2024). Severe COVID-19 and long COVID are associated with high expression of STING, cGAS and IFN-α. Sci. Rep..

