# Supplementary material for: Type I Interferon-Related Gene Expression and Laboratory Abnormalities in Acute Infection Are Associated with Long COVID Symptom Burden
Source: J Clin Med. 2025 Nov 6;14(21):7875. doi: 10.3390/jcm14217875 (PMC12610472; doi:10.3390/jcm14217875)
Supplement: Supplementary file 1 [file jcm-14-07875-s001.zip › jcm-3939176-supplementary.pdf]

Long Covid Pre-Assessment Questionnaire

The purpose of the questionnaire is to provide a means of recording important information about you and your condition.
Please complete this in your own time, ready to discuss at your assessment.
It is important that people who went to hospital, and those who did not, complete the questionnaire.
Where there are multiple choices please circle the one that applies to you.

In section 1 we ask some questions about you. This is because it has become increasingly clear that COVID-19 has a disproportionate impact on some people, such as those living in areas of deprivation, people from Black, Asian and minority ethnic communities (BAME), older people, men, those with a learning disability and others with protected characteristics. We want to do as much as we can to eliminate this inequality. The questions in section 1 will help us to see whether certain groups of people are more affected by Long Covid than others. Knowing this will help us to help you and others more effectively.
You will be asked to share your answers at assessment.

Section 1

Full Name	
NHS number (if known)	
Postcode	
Ethnic Group<br>(circle as appropriate)	White<br>Mixed or multiple ethnic groups<br>Asian or Asian British<br>Black, Black British, Caribbean or African<br>Other ethnic group<br>Prefer not to say
Sex	Female<br>Male<br>Is this the gender you were assigned at birth? Y/N<br>Prefer not to say
Age	
Do you work, or have you worked for the NHS during the COVID-19 pandemic?	Yes / No

SECTION 2

Date of positive swab	/ No test available
Date of onset of symptoms	
Original/acute covid symptoms	
Long covid symptoms	

Previous week / month long covid symptoms	
Date of discharge (for hospital admissions)	/ Not Applicable
Level of respiratory support during acute illness (circle appropriate)	Intubated<br>Enhanced respiratory support (e.g. Continuous Positive Airway Pressure [CPAP], Supplemental oxygen)<br>Not Applicable

SECTION 3

1a<br>1b	Have you made a full recovery or<br>Are you still troubled by symptoms?	Yes / No<br>Yes / No
2<br>2a<br>2b	Are you more breathless now than you were before your COVID illness?<br>Is this more than you would have expected by now?<br>Do you think you are on your way back to full fitness?	Yes / No<br>Yes / No<br>Yes / No
3<br><br>3a<br>3b	Do you feel fatigued (worn out/lacking energy or zest) compared with how you were before your COVID illness?<br>Is this more than you would have expected by now?<br>Do you think you are well on your way back to full fitness?	Yes / No<br><br>Yes / No<br>Yes / No
4	Do you have a cough (different from any cough you may have had before COVID-19)?	Yes / No / Intermittent<br>Had for ……………… weeks/months
5	Do you get any palpitations? (feeling your heart pounding or racing)	Yes / No
6	How is your physical strength - do you feel so weak that it still limiting what you can do (more than you were before your COVID illness)?	Yes / No
7	Do you have any myalgia ('aching in your muscles')?	Yes / No
8	Do you have anosmia ('no sense of smell')?	Yes / No
9	Have you lost your sense of taste?	Yes / No / metallic taste in mouth
10	Is your sleep disturbed (more than it was before you were ill with-COVID)?	Yes / No
11	Have you had any nightmares or flashbacks?	Yes / No
12	Do you have problems with your memory, concentration or decision making?	Yes / No
13	Do you suffer with recurrent fevers?	Yes / No
14	Do you suffer with joint pains?	Yes / No
15	Do you suffer with frequent or severe headaches?	Yes / No
16	Do you experience chest pains?	Yes / No
17	Have you experienced visual disturbance?	Yes / No
18	Are you experiencing new or worse tinnitus?	Yes / No

19	Do you suffer with periods of nausea?	Yes / No
20	Do you have new, more frequent, or more severe rashes?	Yes / No
21	Is your mood low / do you feel down in the dumps / lacking in motivation / no pleasure in anything (circle anything relevant)	Yes / No / Occasionally
22	Do you find yourself feeling anxious/worrying more than you used to?	Yes / No / Occasionally
23	Have you had any significant anxiety, depression or any other mental health problem in the past?	
24	Did you receive treatment for any mental health difficulty you may have experienced?	
25	Have you lost / gained weight (> ½ stone, 3 Kg) since your COVID illness? (circle as appropriate)	Yes / No<br>Lost / gained<br>approx…….lbs/..…kg
25	Do you have headaches / fuzzy head / base of skull pain / brain fog / lack of concentration (circle any relevant)	Yes / No
27	Do you have any throat, restriction, voice, ear, nasal issues?	Yes / No
28	Do you do any exercise?<br>If yes, how many minutes can you manage without adverse effects?..........................	Yes / No
29	Does pacing (planning when, and how long to spend doing something) help with everyday tasks?	Yes / No
30	Do you feel you have been supported by your GP etc?	Yes / No / Any Comments
31	Any other symptoms (list)?	Intermittent / Permanent
32	Any improved symptoms?	
33	Do you pace (planning when, and how long to spend doing something) everything in your life including exercise (if even possible)	Yes/ No
34	Any additional relevant information?	
35	Of the symptoms you have identified, which are most concerning you?	

36	Do you have good and bad days? If so, over the last 14 days how many days would you rate as bad?	

SECTION 4

37	Have you tried any alternative treatments i.e., acupuncture, low histamine diet etc?	Yes / No<br>Please list if 'yes' :
38	Please list any current medication you are taking	
39	Please list any current vitamins, herbal, homeopathic, supplements etc. that you are taking.	
40	If known, what is your approximate **normal** Blood Pressure / Heart Rate / Blood oxygen level (measured by pulse oximeter)	Blood pressure: Heart rate:<br>Blood oxygen level:
41	Pre-existing Medical Conditions	
42	What help do you think you need?	
43	Are you involved in any research (Covid Symptom Study, Kings College London, Oxford etc)?	If yes, please state:

Thank you for completing this questionnaire.
Please ensure you have this to hand for your assessment.
